# Selective crystallization with preferred lithium-ion storage capability of inorganic materials

**DOI:** 10.1186/1556-276X-7-149

**Published:** 2012-02-21

**Authors:** Fei Liu, Shuyan Song, Dongfeng Xue, Hongjie Zhang

**Affiliations:** 1 State Key Laboratory of Rare Earth Resource Utilization, Changchun Institute of Applied Chemistry, Chinese Academy of Sciences, 5625 Renmin Street, Changchun, 130022, People's Republic of China; 2 School of Chemical Engineering, Dalian University of Technology, Dalian, 116024, People's Republic of China

**Keywords:** crystallization, lithium-ion battery, nanowire, hollow structure, nanocomposites.

## Abstract

Lithium-ion batteries are supposed to be a key method to make a more efficient use of energy. In the past decade, nanostructured electrode materials have been extensively studied and have presented the opportunity to achieve superior performance for the next-generation batteries which require higher energy and power densities and longer cycle life. In this article, we reviewed recent research activities on selective crystallization of inorganic materials into nanostructured electrodes for lithium-ion batteries and discuss how selective crystallization can improve the electrode performance of materials; for example, selective exposure of surfaces normal to the ionic diffusion paths can greatly enhance the ion conductivity of insertion-type materials; crystallization of alloying-type materials into nanowire arrays has proven to be a good solution to the electrode pulverization problem; and constructing conversion-type materials into hollow structures is an effective approach to buffer the volume variation during cycling. The major goal of this review is to demonstrate the importance of crystallization in energy storage applications.

## Introduction

Materials crystallized with unique sizes and structures are expected to find various novel applications [1-5]. The discovery of novel materials, processes, and phenomena provides fresh opportunities for the development of innovative systems and devices, which is likely to have a profound impact in areas such as energy, electronics, medicine, and biotechnology [6-12]. Batteries are a major technological challenge in the present society as they are a key method to make a more efficient use of energy [13-15]. Although the current lithium-ion battery (LIB) technology has conquered the portable electronic markets and is still improving, the use of LIB in the powering of plug-in electric vehicles or the storage of renewable energies (wind, solar) is still challenging [16]. The performance of LIB depends essentially on the thermodynamics and kinetics of the electrochemical reactions involved in the electrode materials. During the past decade, extensive efforts have been made to developing advanced batteries with large capacity, high energy and power densities, high safety, long cycle life, fast response, and low cost [17-20]. These developments rely on new ways to prepare electrode materials via eco-efficient processes; achieving these goals will require the inputs of multiple disciplines.

**Figure 1:**
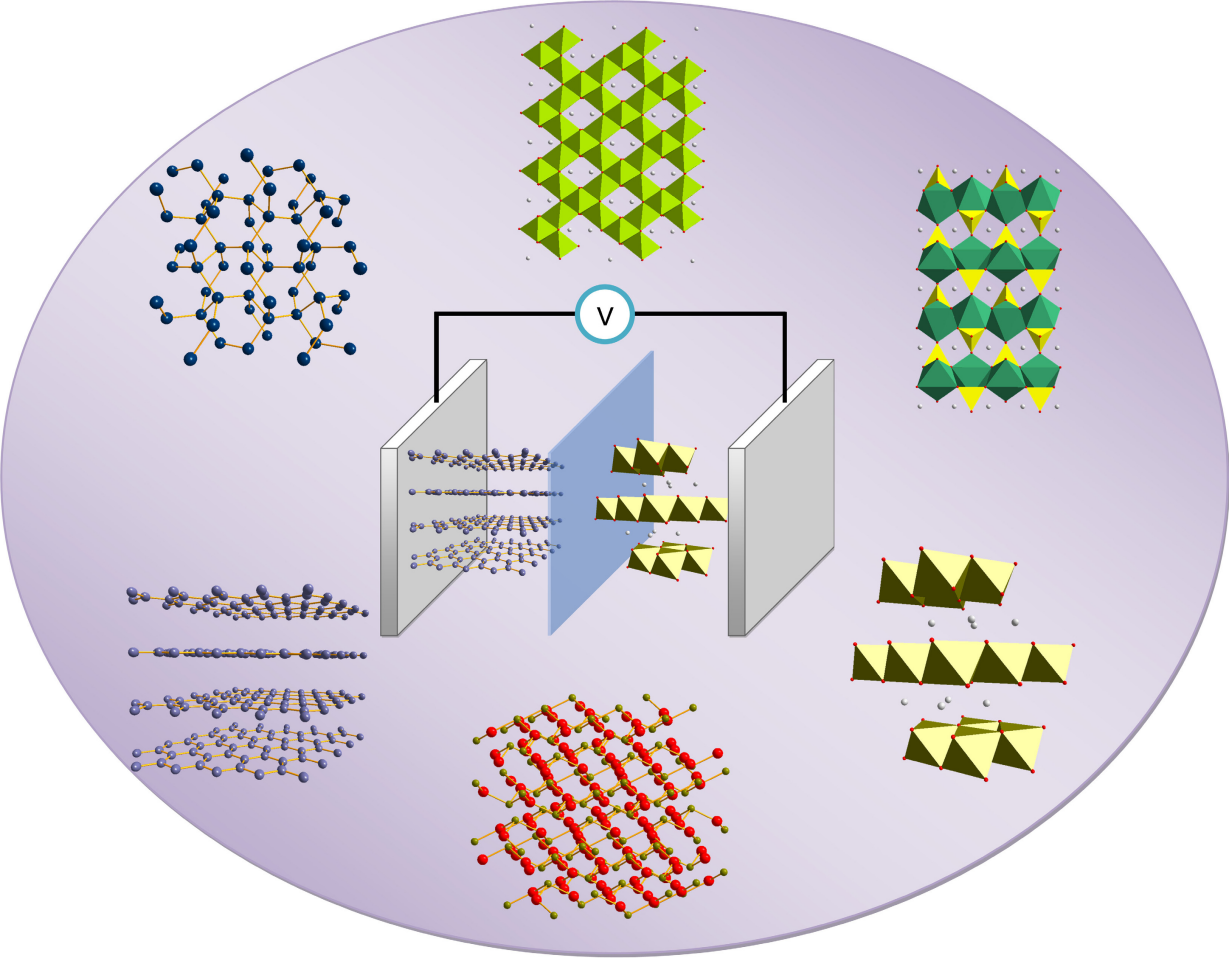
**Schematic illustration of a LIB**. Various materials with different structures can be used for anodes and cathodes.

**Figure 2:**
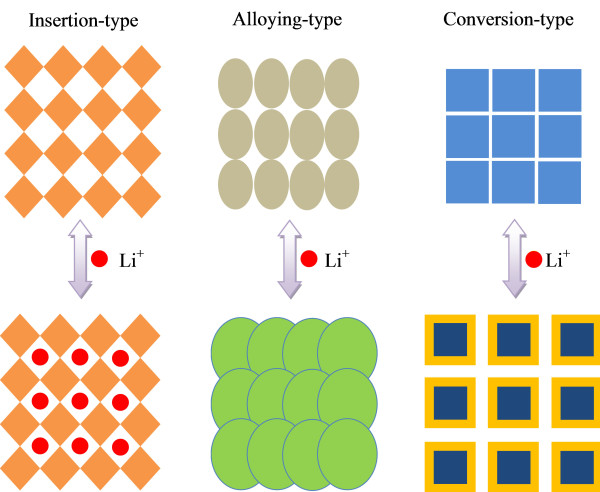
**Schematic illustration of different types of lithium-ion storage materials**.

LIBs comprise four major components: a cathode, anode, separator, and electrolyte (Figure 1). During electrochemical reactions, lithium ions move from the cathode to the anode through the separator and the electrolyte or vice versa. LIB electrode materials can be classified into three groups depending on their reaction mechanisms with lithium ion [21-23], as shown in Figure 2: (1) insertion/extraction reaction mechanism that involves the insertion and extraction of Li into and from the lattice, (2) Li-alloy reaction mechanism, and (3) conversion reaction mechanism that involves the formation and decomposition of Li oxide (Li_2_O), accompanying the reduction and oxidation of metal nanoparticles. These three reaction mechanisms are displayed as follows:

• Insertion reaction mechanism:

(1) $$\text{M}{\text{O}}_{\text{x}}+\text{ yL}{\text{i}}^{+}+\text{ y}{\text{e}}^{-}↔\text{L}{\text{i}}_{\text{y}}\text{M}{\text{O}}_{\text{x}}$$

• Li-alloy reaction mechanism:

(2) $${\text{M}}_{\text{x}}{\text{O}}_{\text{y}}+\text{ 2yL}{\text{i}}^{+}+\text{ 2y}{\text{e}}^{-}\to \text{xM}+\text{yL}{\text{i}}_{\text{2}}\text{O}\;\left(\text{e}.\text{g}.,\text{ Sn}{\text{O}}_{\text{2}}\right)$$

(3) $$\text{M}+\text{zL}{\text{i}}^{+}+\text{ ze}-↔\text{L}{\text{i}}_{\text{z}}\text{M}$$

• Conversion reaction mechanism:

(4) $${\text{M}}_{\text{x}}{\text{O}}_{\text{y}}+\text{ 2yL}{\text{i}}^{+}+\text{ 2y}{\text{e}}^{-}↔\text{xM}+\text{yL}{\text{i}}_{\text{2}}\text{O}$$

Insertion-type materials containing cobalt are the most studied cathodes for LIB [21]. They show high stability in a high-voltage range; however, cobalt has limited availability in nature and is toxic, which is a tremendous drawback for mass manufacturing. Manganese offers a low-cost substitution with a high thermal threshold and excellent rate capabilities but limited cycling behavior [21]. Olivines are nontoxic and have a moderate capacity with low fade due to cycling, but their conductivity is quite low [24]. Alloy anodes have high capacities but show a dramatic volume change in charging and discharging, resulting in poor cycling behavior [25], a similar problem also found in conversion-type materials [26].

Nanostructuring electrode materials has been proven to be an effective strategy to alleviate these above problems [27-31]. There are several advantages associated with the development of nanomaterials for LIBs [27], which include (1) better accommodation of the strain of lithium insertion/removal, improving cycle performance; (2) new reactions which are not possible in bulk materials may happen; (3) better electrode/electrolyte contact, and (4) short path lengths for electron and Li^+ ^transport. Here, we summarize recent scientific research and development of LIB electrode materials upon novel nanoscience and nanotechnology progresses. The focus is on research activities toward the selective crystallization of inorganic materials with preferred shapes, sizes, and structures, which can influence ionic diffusion and transport, electron transfer, surface/interface interaction, and the electrochemical reactions. The effect of selective crystallization on the LIB performances of electrode materials is discussed in detail according to different Li storage mechanisms. The current review shows that the selective crystallization route plays a predominant role in the development of next-generation LIBs.

### Insertion-type materials

Insertion-type materials involve most cathode materials and some anode materials (such as graphite, Li_4_Ti_5_O_12_, and TiO_2_). The first generation of LIB uses LiCoO_2 _and graphite as the positive and negative electrodes; the redox operation of both versus lithium is based on intercalation reactions. Alternative materials such as LiMn_2_O_4_, LiFePO_4_, and Li_4_Ti_5_O_12 _have also reached the market at different levels, bringing about incremental improvements in performance [32]. Nonetheless, all these materials have intrinsic capacity limitations, which are derived from their redox mechanisms and structural aspects, i.e., the intrinsic redox activity of the transition metals and the changes the crystal structure can withstand. Such limitation handicaps the device in terms of energy density. Power density of these cathode materials with bulk sizes is also generally low due to the high level of polarization at high charge/discharge rates [32]. Therefore, the selective crystallization approach was introduced to overcome these shortcomings by decreasing diffusion paths for mass transport and increasing the surface area for charge transfer.

**Figure 3:**
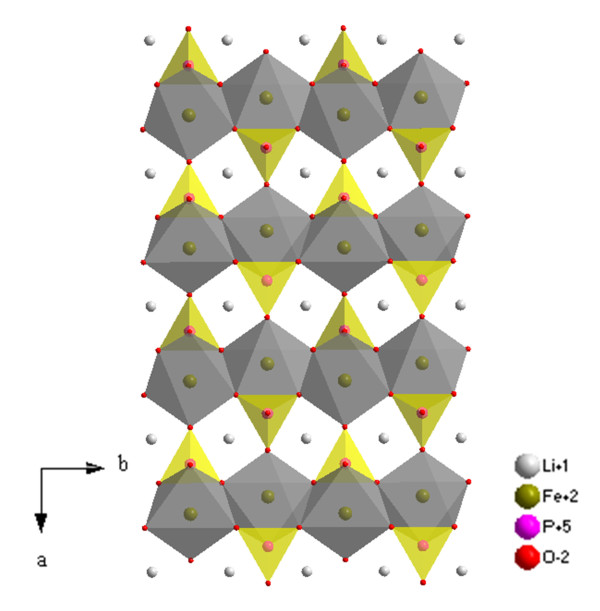
**The crystal structure of olivine LiFePO_4 _in projection along [001]**.

The characteristic structure of insertion-type materials is an ionic diffusion path. It is clear that morphological control of nanocrystalline materials is significantly important. The selective exposure of surfaces normal to the most facile pathway for lithium-ion conduction is preferred, and the length of particles along the ionic diffusion direction should be decreased since it can enhance electrochemical performance by reducing transport path lengths as well as enhance the electrode/electrolyte contact [33]. For example, in an olivine structure where ionic diffusion paths are along the b-axis (Figure 3), a smaller dimension along the ionic diffusion direction can be obtained via the realization of three types of morphologies: (1) zero-dimensional spherical nanoparticles; (2) one-dimensional (1D) nanorods oriented with long axes along the a or c direction; or (3) two-dimensional (2D) nanoplates with a (101) basal plane.

**Figure 4:**
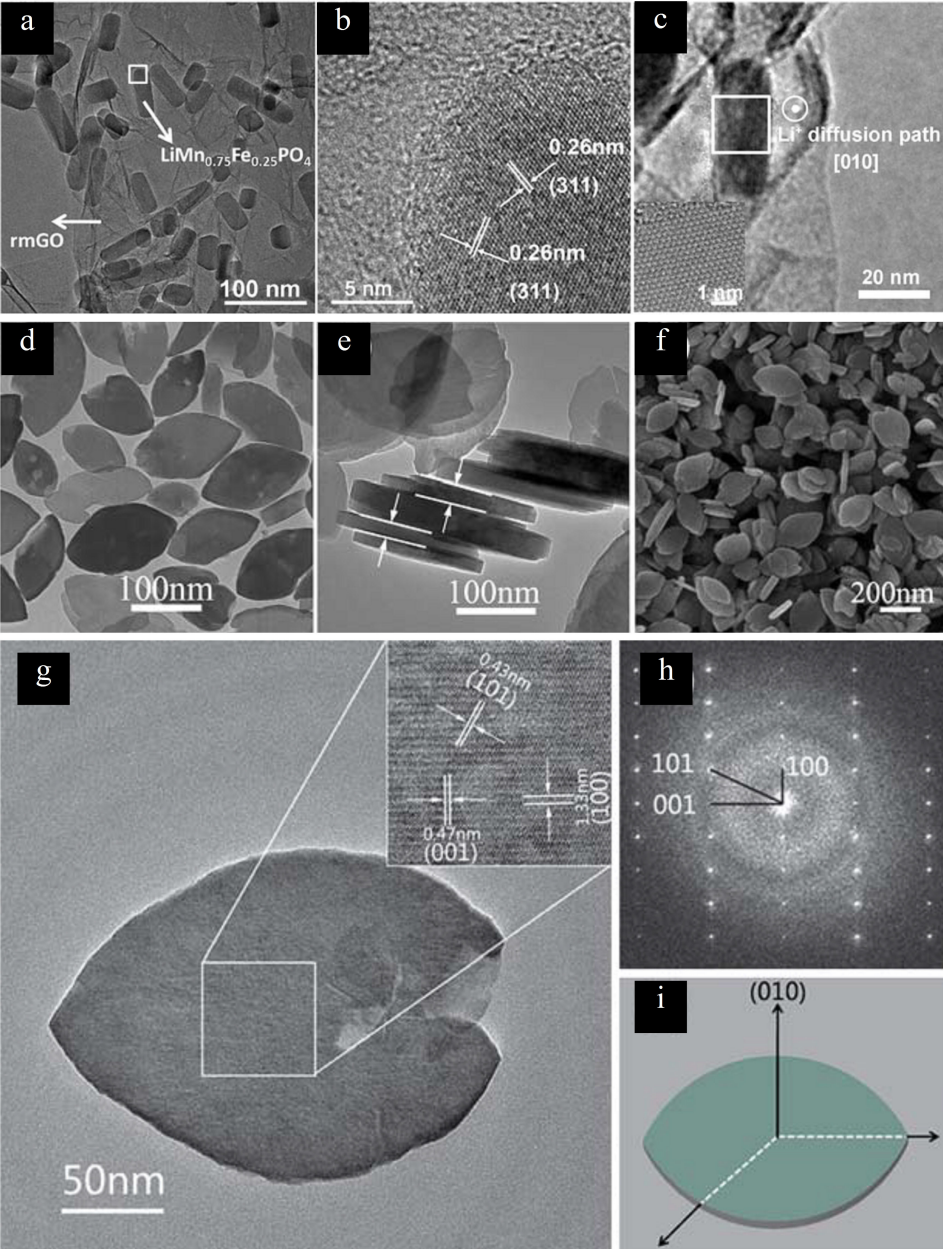
**1D nanorods of LiMn_1-x_Fe_x_PO_4 _and 2D nanoplates of LiFePO_4_**. (**a-c**) Transmission electron microscopy (TEM) images of 1D nanorods of LiMn_1-x_Fe_x_PO_4 _with a radial [010] direction [36]. Copyright Wiley-VCH Verlag Gmbh & Co. KGaA. Reproduced with permission. (**d-i**) TEM and scanning electron microscopy (SEM) images, selected area electron diffraction (SAED) pattern, and structure illustration of 2D nanoplates of LiFePO_4 _with large exposure of (010) face. Adapted from [37] and reproduced with the permission of the Royal Society of Chemistry.

Based on this idea, nanoparticles of LiFePO_4 _olivine materials have been synthesized by conventional solid-state synthetic methods and polyol synthesis [34,35]; 1D nanorods of LiMn_1-x_Fe_x_PO_4 _with a radial [010] direction can be selectively crystallized by a controlled hydrolysis method (Figure 4a, b, c) [36]. Also, by employing solvothermal synthesis, 2D nanoplates of LiFePO_4 _with large exposure of (010) face can be obtained (Figure 4d, e, f, g, h, i) [37]. These nanostructured olivine electrodes exhibited better rate performances than bulkier materials due to smaller diffusion length, indicating that selective crystallization is an effective way to obtain high-electrochemical-performance materials.

**Figure 5:**
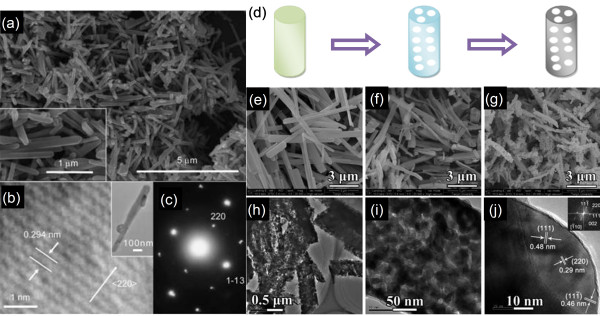
**Single-crystalline LiMn_2_O_4 _nanorods; porous LiMn_2_O_4 _nanorod formation process; MnC_2_O_4_, porous Mn_2_O_3_; and porous LiMn_2_O_4 _nanorods**. (**a**) SEM and (**b**) TEM images and (**c**) SAED pattern of single-crystalline LiMn_2_O_4 _nanorods fabricated using single-crystalline MnO_2 _nanorods as precursor. Adapted from [40] and reproduced with the permission of the American Chemical Society. (**d**) Illustration of the porous LiMn_2_O_4 _nanorod formation process. SEM micrographs of (**e**) MnC_2_O_4 _nanorods, (**f**) porous Mn_2_O_3 _nanorods, and (**g**) porous LiMn_2_O_4 _nanorods. (**h**, **i**) TEM images of the porous LiMn_2_O_4 _nanorods. (**j**) High-resolution TEM (HRTEM) image and the corresponding fast Fourier transform diffraction pattern (inset) of a single nanoparticle in the porous nanorods. Adapted from [41] and reproduced with the permission of the Royal Society of Chemistry.

The spinel-type LiMn_2_O_4 _has also attracted continued interest. LiMn_2_O_4 _is favorable for its safety and intrinsic rate capability and has been established as cathode materials for electric vehicle applications [38]. However, the gradual capacity loss due to Jahn-Teller distortion of Mn^3+ ^and Mn dissolution in the electrolyte has hindered its application [39]. A solution to this problem is selective crystallization of nanostructured spinels such as LiMn_2_O_4 _nanorods and nanowires. In a nanostructured spinel, the phase-boundary energy dramatically changes with the particle size, which can promote the solubility and solid solution properties and thus changes the kinetics and thermodynamics of Li-insertion reactions. Freestanding, single-crystalline LiMn_2_O_4 _nanorods have been fabricated using single-crystalline MnO_2 _nanorods as precursor via a simple solid-state reaction [40], as shown in Figure 5. LIB testing showed that LiMn_2_O_4 _nanorods can deliver a capacity of 100 mAh g^-1 ^at a high current density of 148 mA g^-1 ^with high reversibility and good capacity retention; after 100 cycles, more than 85% of the initial capacity was maintained. An extended method to prepare porous LiMn_2_O_4 _nanorods has also been reported, using porous Mn_2_O_3 _nanorods resulting from the thermal decomposition of MnC_2_O_4 _as precursor [41]. The as-synthesized porous nanorods exhibited high rate capability and cyclability. An initial discharge capacity of 105 mAh g^-1 ^was obtained at a 10 C rate, and capacity retention of about 90% was obtained after 500 cycles. The authors attributed this durable, high rate capability to the unique, porous 1D nanostructure that gave rise to fast Li-intercalation kinetics and good structural stability for the spinel electrodes.

**Figure 6:**
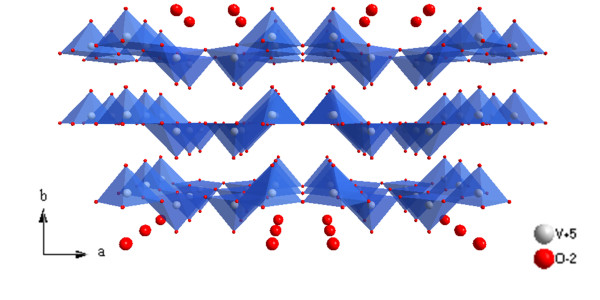
**The crystal structure of orthorhombic V_2_O_5 _in projection along [001]**.

**Figure 7:**
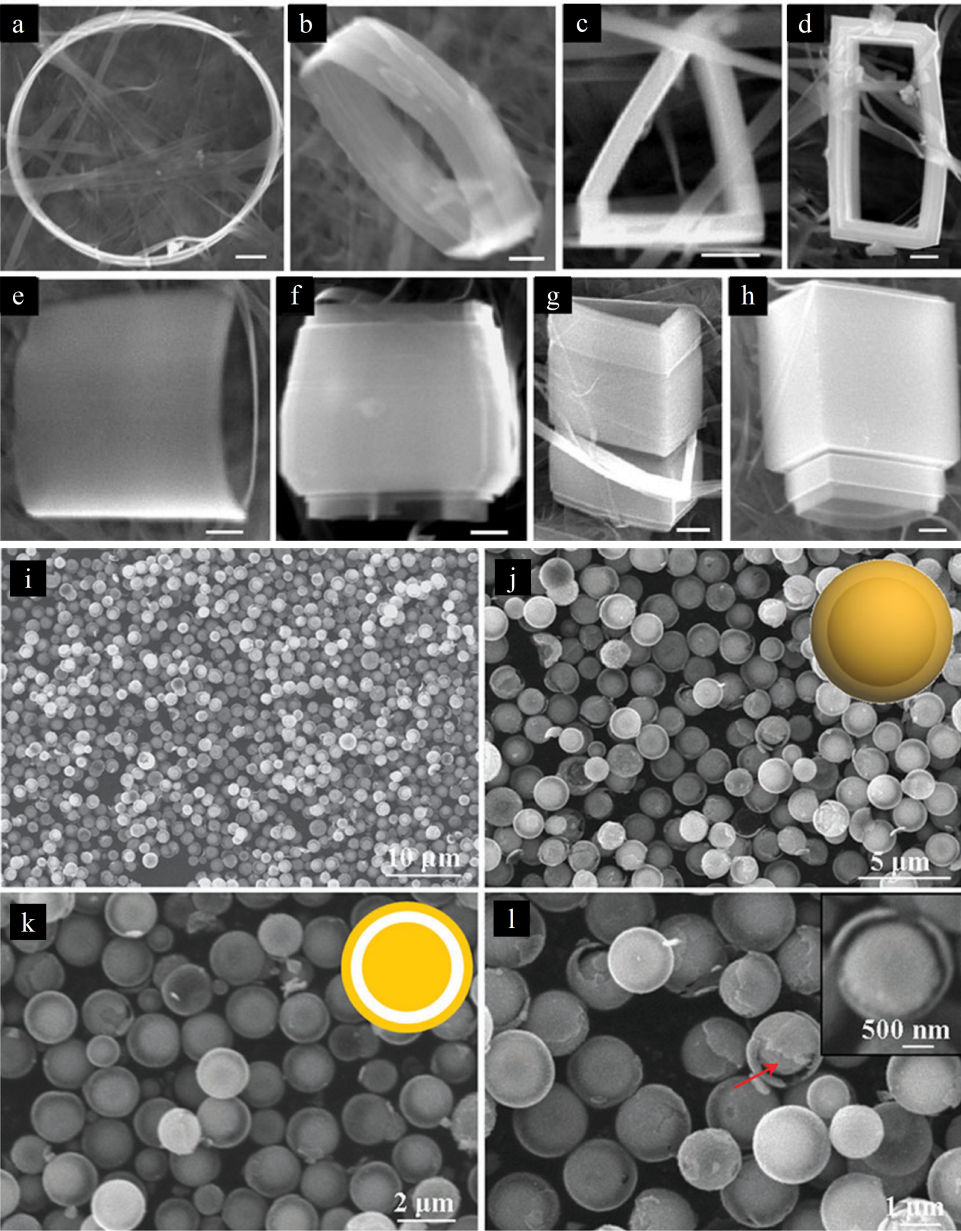
**Single-crystalline V_2_O_5_·xH_2_O nanorings and microloops, and yolk-shell V_2_O_5 _microspheres**. (**a-h**) SEM images of single-crystalline V_2_O_5_·xH_2_O nanorings and microloops [43]. (**i-l**) SEM images of yolk-shell V_2_O_5 _microspheres. The insets of (j) and (k) show the schematic structure of yolk-shell V_2_O_5 _microspheres. Adapted from [44] and reproduced with the permission of the Royal Society of Chemistry.

Vanadium oxide (V_2_O_5_) is a typical intercalation compound as a result of its layered structure; orthorhombic, crystalline V_2_O_5 _consists of layers of VO_5 _square pyramids that share edges and corners (Figure 6). For LIB applications, V_2_O_5 _offers the essential advantages of low cost, abundant source, easy synthesis, and high energy densities [42]. The crystallization of V_2_O_5 _into specific nanostructures also plays an important role in improving the electrochemical performance of rechargeable LIBs. For controlling the nanostructure of V_2_O_5_, Liu and Xue reported a solution route to fabricate single-crystalline V_2_O_5_·xH_2_O nanorings and microloops (Figure 7a, b, c, d, e, f, g, h) [43], the formation of rolling structures are caused by the cation-induced asymmetric strain on layered-structure V_2_O_5_·xH_2_O nanobelts. This work demonstrates that the novel nanoring structure, which has been observed previously only for polar surface-dominated structure materials, can also form in compounds without anion- and cation-terminated surfaces. This proposed cation-induced strategy extends the existing formation mechanism of nanorings and can be applied to other materials. Recently, Liu and Xue also reported a scalable, highly reproducible, and template-free process to crystallize a yolk-shell V_2_O_5 _microsphere cathode material (Figure 7i, j, k, l) [44]. This featured material has a high specific capacity (280 mAh g^-1^) in the initial discharge process and excellent retention of the initial capacity after 30 cycles. During the whole cyclic process, the Coulombic efficiency steadily kept the values higher than 95%. The enhanced electrochemical performance is closely related to the selectively crystallized yolk-shell microstructure. The hollow-structured feature facilitates the electrolyte transport, and the small granularity and cavity among individual nanoparticles (porous shell) can effectively prevent the amorphization of the electrode, which is the main cause of the capacity fading of V_2_O_5 _during cycling.

Besides increasing capacity, a high cell voltage resulting from a high (cathode) and low (anode) standard redox potential of the respective electrode redox reaction can also greatly improve the energy density of LIB. Due to the redox potential of an intercalation compound which mainly tracks the iono-covalency of the metal-X bonding, selective crystallization of cathode materials with fluorine substitution appears to be quite an attractive route to increase the material redox voltage as F is very electronegative [16]. Recently, Barpanda et al. reported a polyanionic material that crystallizes in the triplite structure Li(Fe_1-δ_Mn_δ_)SO_4_F [45]. An open-circuit voltage of 3.9 V has been achieved, exceeding that of LiFePO_4 _by 450 mV. Also, this new triplite phase is capable of reversibly releasing and reinserting 0.7 to 0.8 Li ions with a volume change of only 0.6% (compared with 7% and 10% for LiFePO_4 _and LiFeSO_4_F, respectively), to give a capacity of 125 mAh g^-1^. Such a material could become a promising cathode to replace LiFePO_4_. These new types of polyanionic compound, having the triplite structure, provide valuable information in the search for even better cathode materials.

In the case of anode materials, the formation of a solid-electrolyte-interface (SEI) layer on which metallic lithium is deposited during a fast charge of the battery should also be a concern. The dendrites can grow to short-circuit the battery and ignite the electrolyte. Therefore, safety concerns have led to a search for anode materials having a redox couple in the range of 1.0 to 1.5 eV below the Fermi energy of lithium. The spinel Li_4_Ti_5_O_12 _is reported to be a stable anode operating on the Ti(IV)/Ti(III) redox couple located at 1.5 V versus Li^+^/Li. It is capable of a fast charge and a long cycle life because no SEI layer is formed [46-48]. However, it has a low specific capacity (approximately 140 mAh g^-1^), and the high redox potential (1.5 V) reduces the energy density of a cell using this anode. On the basis of these considerations, different niobium-based oxides such as KNb_5_O_13 _and K_6_Nb_10.8_O_30 _have been investigated, which exhibit a reversible Li insertion toward the targeted voltage range of 1.0 to 1.5 V versus Li^+^/Li [49,50]. More recently, the mixed titanium-niobium oxides such as TiNb_2_O_7 _have been selectively crystallized as the anode for lithium batteries with some similar electrochemical properties [51,52]. Notably, carbon-coated TiNb_2_O_7 _gives a reversible specific capacity of approximately 285 mAh g^-1 ^cycled between 1.0 and 2.5 V versus Li+/Li with a Coulombic efficiency over 98% [52]. These new-type anodes provided promising candidates for batteries with high rate, high cycle life, and better safety.

From the above discussion, it is evident that selective crystallization of insertion-type materials into nanosized particles and shapes with specific facets can effectively enhance the lithium-ion diffusion rate and improve their cycling performance; these improvements are all closely related to the crystal structure and reaction mechanisms of this kind of materials. As an alternative route, tuning the crystal structure by selective atom substitution can also improve the energy density of LIB by increasing the cell voltage.

### Alloying-type materials

**Figure 8:**
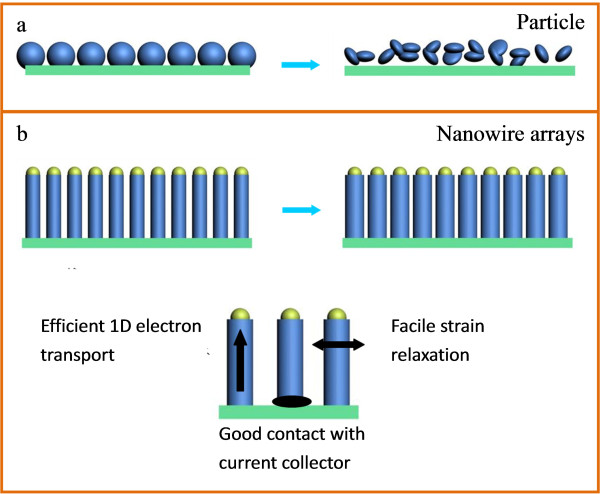
**Schematic illustration of structure changes during the electrochemical cycling process**. (**a**) Si particles and (**b**) Si nanowire arrays.

Some main-group elements (e.g., Si, Ge, Sn, Al, Bi, Zn, and Sb) can alloy with lithium at a low potential. Representatively, Si and Sn can form lithium alloys with Li compositions up to Li_4.4_M, giving theoretical specific capacities as high as 4,200 and 993 mAh g^-1^, respectively [53-55]. Unfortunately, huge volume changes occur during the electrochemical lithiation/delithiation process [56]. For example, a volume expansion on the order of 400% occurred during the formation of Li_4.4_Si alloy, which causes cracking and eventual pulverization of the electrode and results in rapid capacity decline (Figure 8a).

**Figure 9:**
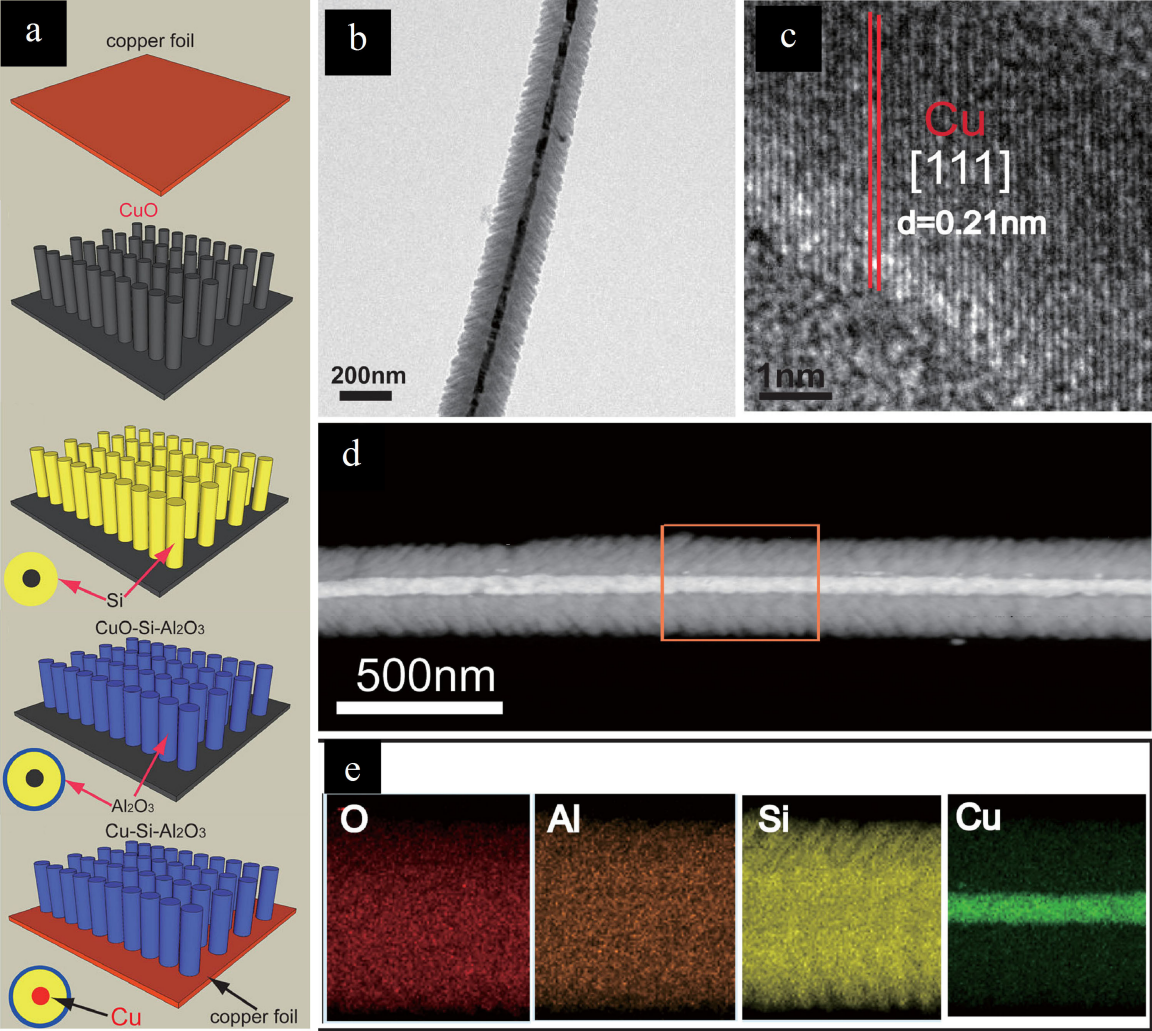
**Cu-Si and Cu-Si-Al_2_O_3 _nanocables and their fabrication**. (**a**) Schematic diagram showing the fabrication of Cu-Si and Cu-Si-Al_2_O_3 _nanocables. (**b**) TEM and (**c**) HRTEM images of a Cu-Si-Al_2_O_3 _nanocable. (**d**) Annual dark-field TEM image and (**e**) corresponding elemental mappings of O, Al, Si, and Cu for the Cu-Si-Al_2_O_3 _nanocable [53]. Copyright Wiley-VCH Verlag Gmbh & Co. KGaA. Reproduced with permission.

As a solution against the volume change problem, selective crystallization of alloy materials into different nanoforms has been suggested because the toughness and adhesion effect within grain boundaries may increase at nanoscale and nanostructured alloys are more flexible to accommodate the strain induced by volume variations [57]. Yang et al. first reported that nanosize Sn electrodes can exhibit much better cycling performance than bulk Sn electrodes due to a smaller absolute volume change [58]. Recently, 1D nanowires of alloy materials have received more attention. Chan et al. reported that Si nanowire arrays grown directly on a current collector exhibited stable cycle performances, because the nanowires were not pulverized or broken due to facile strain relaxation of the nanowire geometry (Figure 8b) [59]. Also, the efficient 1D electron transport and good contact with current collector also contribute to their improved LIB performance. Cao et al. presented a concept of using Cu-Si and Cu-Si-Al_2_O_3 _nanocable arrays directly grown from the current collector as LIB anodes (Figure 9) [60]. The conductive Cu cores that are anchored to the copper foil act as both current collectors and structural reinforcements for the Si shell of the nanocables. The outer surface of the nanocables is readily modified by an additional sheath of Al_2_O_3_, which provides a stable Si/electrolyte interface. Both nanocables show excellent electrochemical performance including high specific capacity and cycling stability (1,890 and 1,820 mAh g^-1 ^under a discharge and charge current density of 0.3 A g^-1^). After coating with Al_2_O_3 _sheaths, the Cu-Si-Al_2_O_3 _nanocables showed a remarkable high rate capability and delivered a capability of 1,140 mAh g^-1 ^at 7 A g^-1^.

**Figure 10:**
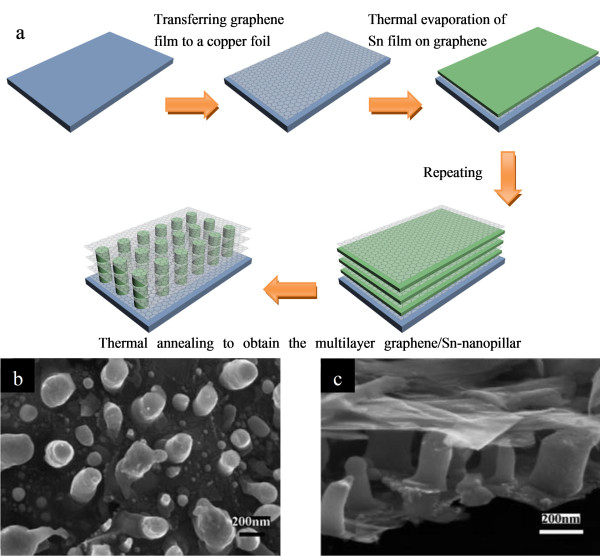
**Graphene/Sn-nanopillar composite and its preparation procedure**. (**a**) Schematic illustration of the graphene/Sn-nanopillar composite preparation procedure. (**b**, **c**) SEM images of graphene/Sn-nanopillar composite. Adapted from [60] and reproduced with the permission of the Royal Society of Chemistry.

Carbon protection has been proven to be an effective route to enhance the cycle stability of alloying-type electrode materials [61]. Because of their high capacity and wide availability, Si-based nanocomposites are among the most attractive anode materials, and much progress has been made in this regard. Some recent examples include graphene-grafted Si nanoparticles [62] and Si nanowires with carbon nanotube coatings [63]. Notably, selective crystallization of Si on the hierarchical carbon spheres with irregular channels has been reported. The hierarchical composite possesses an interconnected, aperiodic, porous network with internal channels, enabling high accessibility of the active Si for fast lithiation. Large Si volume changes can be accommodated by the particle's internal porosity. A reversible capacity of 1,950 mAh g^-1 ^and a stable performance are attained [64]. In the case of tin-carbon composite materials, porous structures including Sn nanoparticles confined in hollow carbon capsule and coaxial SnO_2_@C hollow spheres proved to be promising anode candidates for highly reversible lithium storage [65,66]. Recently, Sn nanopillar arrays embedded between graphene sheets were assembled using a conventional film deposition and annealing process (Figure 10) [67]. The as-formed three-dimensional (3D) multilayered nanostructure can be directly used as an anode material without adding any polymer binder and carbon black. This composite showed high reversible capacity (714 mAh g^-1^) and excellent cycling performance at a high current density of 5 A g^-1 ^and demonstrated that a highly functional nanocomposite can also be fabricated by employing conventional top-down manufacturing methods and self-assembly principles.

**Figure 11:**
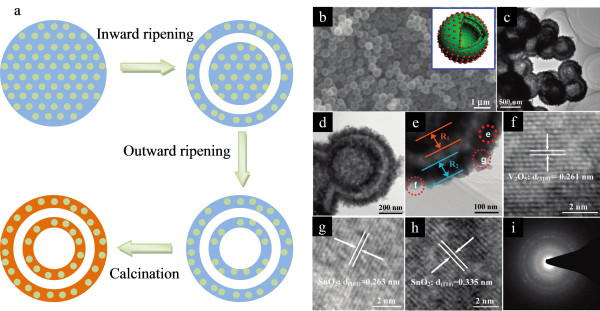
**V_2_O_5_-SnO_2 _double-shelled nanocapsules and their formation process**. (**a**) Schematic presentation of the formation process of V_2_O_5_-SnO_2 _double-shelled nanocapsules based on Ostwald ripening. (**b**) SEM image of V_2_O_5_-SnO_2 _double-shelled nanocapsules. Inset shows the schematic structure of these double-shelled nanocapsules. (**c-e**) TEM images indicate that the porous shell consists of a great deal of nanocrystals. (**f-h**) HRTEM images revealing lattice planes of the V_2_O_5 _matrix and SnO_2 _nanocrystals. (**i**) SAED pattern taken from individual nanocapsules which shows that these nanocapsules are polycrystalline. Adapted from [62] and reproduced with the permission of the American Chemical Society.

To tackle the volume change problem, selective crystallization of alloying-type materials into specific structures has also been employed. Some peculiarly designed nanoscale electrodes, such as hollow nanostructures, have more free space and can endure larger stresses [68]. For example, Liu et al. successfully designed a template-free route to fabricate double-shelled SnO_2_-V_2_O_5 _nanocapsules [69]. The formation mechanism of these double-shelled, hollow nanocapsules is a combination of both inward and outward Ostwald ripening processes, which is shown in Figure 11a. Ostwald ripening firstly took place at the surface of these solid nanospheres, which results in the void formation between two layers. Following this inward ripening process, the solid core of nanospheres ripened outward, leading to a double-shelled V_2_O_3_-SnO_2 _hollow structure. Finally, double-shelled V_2_O_5_-SnO_2 _nanocapsules were obtained by the calcination of these V_2_O_3_-SnO_2 _nanocapsules in open air. Figure 11b, c, d, e, f, g, h, i shows the structural characterizations of these composite nanocapsules. The double-shelled structure is obvious from these pictures, and SnO_2 _crystallines are homogenously distributed in the V_2_O_5 _matrix. These V_2_O_5_-SnO_2 _hollow nanocapsules showed a large reversible capacity of 947 mAh g^-1 ^as an anode material and good cycling stability (can deliver a reversible capacity of 673 mAh g^-1 ^after 50 cycles). The reversible capacity as an anode can be further improved by increasing the content of SnO_2 _in nanocomposites. With 15 wt.% SnO_2 _content, the first discharge capacity can reach 1,776 mAh g^-1^; after 20 cycles, the reversible discharge capacity maintains 1,046 mAh g^-1 ^without obvious capacity fading except for the first cycle. The excellent capacity retention was largely attributed to the large free space between the shells which can effectively accommodate the volume variation. It is worth noting that tin-based anodes have already been applied in the commercialized rechargeable LIBs. Therefore, the development of alloy anodes has opened a new avenue in the fabrication of advanced LIBs.

### Conversion-type materials

Interstitial-free, 3D transition-metal oxides (M_x_O_y_, M = Fe, Co, Ni, Mn, Cu, etc.) are capable of incorporating more than one Li per 3D metal, hence giving high Li storage capacities [70]. The Li storage mechanism of the M_x_O_y _differs from the Li-intercalation and Li-alloying mechanisms. Transition-metal oxides are reduced to metal in the lithiation process (Equation 4). During the first reduction of the metal oxide, highly reactive metallic nanodomains embedded in a Li_2_O matrix can be generated *in situ*, which contributed to the reversibility of this reaction [70]. Based on this mechanism, reversible lithium storage proceeds more easily with the nanostructured oxides. Therefore, selective crystallization of transition-metal oxides has a significant role for their LIB performances. Similar to the Li-alloying process, the conversion reaction leads to volume variation upon the electrochemical cycling. Conceptually, approaches such as constructing hollow structures or nano-compositing are applicable to conversion-type anode materials as well.

**Figure 12:**
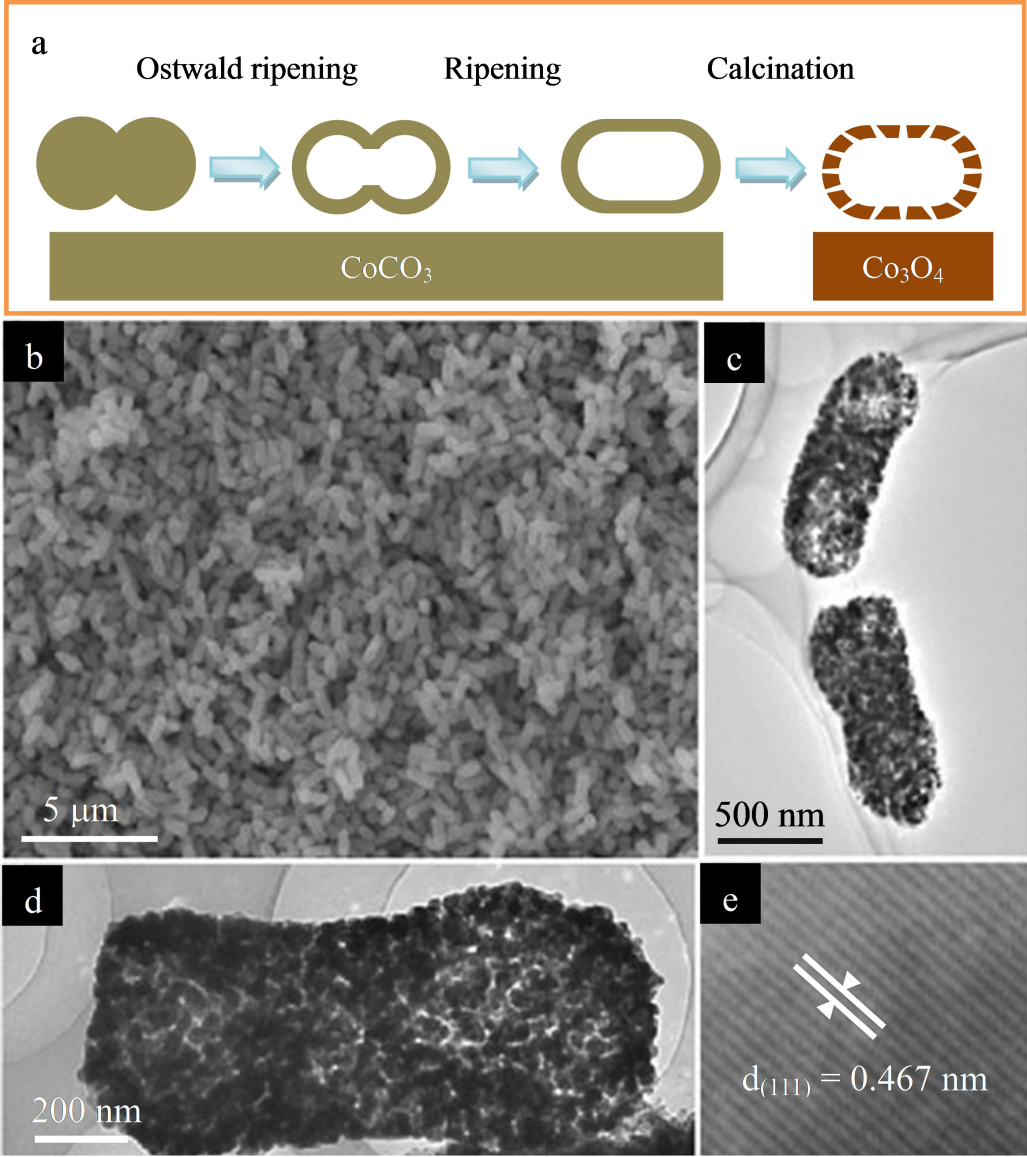
**Anisotropic porous Co_3_O_4 _nanocapsules and their evolution process**. (**a**) Schematic presentation of the evolution process of anisotropic Co_3_O_4 _porous nanocapsules. (**b**) Low-magnification SEM image showing that these nanocapsules are nearly monodisperse. (**c**) Low-magnification TEM image showing the porous shell of anisotropic Co_3_O_4 _nanocapsules. (**d**) High-magnification TEM image exhibiting a single, porous Co_3_O_4 _nanocapsule. (**e**) HRTEM image of individual nanocrystals revealing the (111) lattice plane. Adapted from [64] and reproduced with the permission of the Royal Society of Chemistry.

Recently, Liu et al. reported a novel self-templated method to fabricate anisotropic Co_3_O_4 _porous and hollow nanocapsules from CoCO_3 _precursors [71]. The selective crystallization is based on the inside-out Ostwald ripening process. Figure 12a illustrates the transformation process from CoCO_3 _precursors to anisotropic Co_3_O_4 _porous and hollow nanocapsules. During the solvothermal process, two spherical CoCO_3 _colloids aggregated and fused together under the driving force of the magnetic dipole interaction between these spherical precursors, forming anisotropic, dumbbell-like structures. Subsequently, these dumbbell-like colloids underwent a ripening process to form nanorods, and the subsequent heat treatment of these CoCO_3 _nanorods led to the formation of Co_3_O_4 _shells; the generation of nanoporous can be attributed to the release of CO_2 _from the CoCO_3 _nanocrystals along different directions. Figure 12b, c, d shows the surface morphology and microstructure of CoCO_3 _precursors and corresponding Co_3_O_4 _porous and hollow nanocapsules; these nanocapsules have nanoporous shells with dense nanopores. The LIB testing of these Co_3_O_4 _porous nanocapsules showed that this kind of structure exhibited superior performances with good cycle life and high capacity, at a discharge/charge current density of 110 mA g^-1^; these anisotropic Co_3_O_4 _porous and hollow nanocapsules showed a high reversible capacity of 1,018 mAh g^-1^, and a capacity of 1,000 mAh g^-1 ^can be obtained after 20 cycles. These high performances can be attributed to their porous and hollow nanostructures and small size of building blocks. Self-supported Co_3_O_4 _mesoporous nanowires directly grown on a Ti substrate as current collector were prepared using a template-free ammonia evaporation-induced method [72]. Due to the novel hierarchical structure that favors electrolyte diffusion and electric contact, the mesoporous nanowire array delivered a first discharge capacity of 1,124 mAh g^-1 ^and maintained a stable capacity of 700 mAh g^-1 ^after 20 cycles, and a considerable capacity of 450 mAh g^-1 ^can be obtained at a high rate of 20 C.

**Figure 13:**
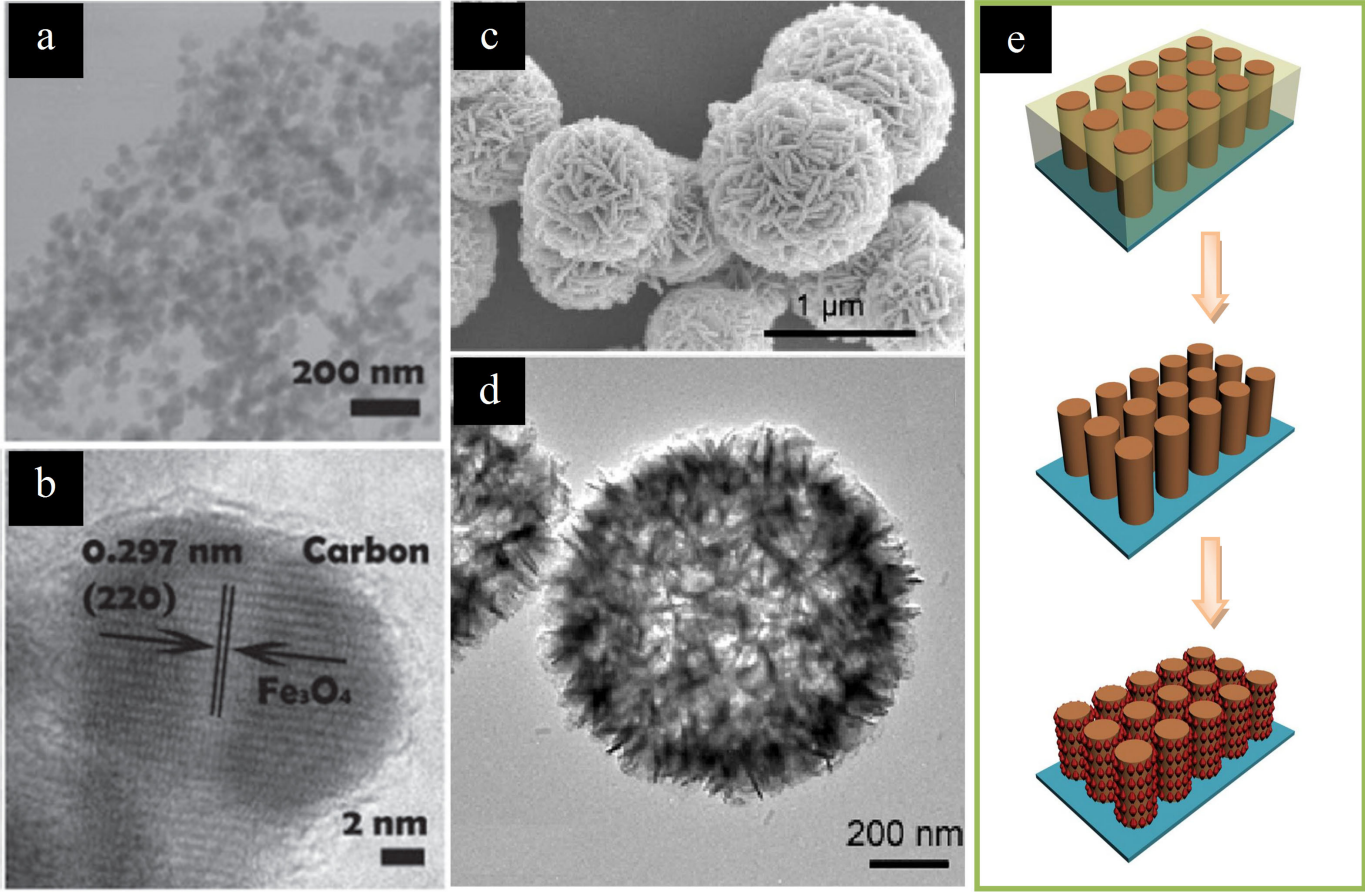
**Fe_3_O_4 _nanoparticles, α-Fe_2_O_3 _hollow spheres, and the two-step electrode design**. (**a**, **b**) TEM images of Fe_3_O_4 _nanoparticles encapsulated within carbon shells anchoring on RGO sheets. Adapted from [66] and reproduced with the permission of the Royal Society of Chemistry. (**c**) SEM and (**d**) TEM images of α-Fe_2_O_3 _hollow spheres with sheet-like subunits. Adapted from [67] and reproduced with the permission of the American Chemical Society. (**e**) Illustration of a two-step electrode design consisting the electrochemically assisted template growth of Cu nanorods onto a current collector followed by the electrochemical plating of Fe_3_O_4_.

Compared to Co, Fe is of lower cost, lower toxicity, and higher abundance, rendering iron oxides as more attractive anode materials. In particular, the spinel Fe_3_O_4 _demonstrates high electronic conductivity and is suitable for potential high-power application. A 3D composite has been constructed by selectively crystallizing Fe_3_O_4 _nanoparticles encapsulated within carbon shells onto reduced graphene oxide (RGO) sheets (Figure 13a, b) [73], which exhibited enhanced anode performances in LIBs with a specific capacity of 842 mAh g^-1 ^and superior recycle stability after 100 cycles; these can be attributed to the unique 3D structure of the composite; the 2D layered structure of RGO combined with the close structure of carbon shells provided a rigid and highly conductive matrix for Fe_3_O_4 _nanoparticles. Besides Fe_3_O_4_, Fe_2_O_3 _has also attracted much interest. For example, selective crystallization of α-Fe_2_O_3 _hollow spheres with sheet-like subunits can be achieved by a quasiemulsion-templated method (Figure 13c, d) [74]. Quasiemulsion microdroplets of glycerol were dispersed in water to serve as soft templates for the deposition of the α-Fe_2_O_3 _shell. When tested as anode materials for LIBs, these α-Fe_2_O_3 _hollow spheres showed a high reversible capacity of 710 mAh g^-1^, even after 100 cycles. A two-step electrode design consisting of the electrochemically assisted template growth of Cu nanorods onto a current collector followed by electrochemical plating of Fe_3_O_4 _was also proposed (Figure 13e) [75]. Using such electrodes, a factor of six improvement in power density over planar electrodes while maintaining the same total discharge time can be achieved. The capacity at the 8 C rate was 80% of the total capacity and was sustained over 100 cycles. Such findings will help pave the way for the application of conversion reaction electrodes in LIB.

The use of transition-metal oxides as conversion-type electrodes holds the promise of higher energy density and wealth of compounds, but capacity fading needs to be overcome before practical use in LIB; bulk electrodes fail within a few charge/discharge cycles due to the large volumetric change that occurs during lithiation and delithiation. Selective crystallization into specific structures and composites can have a major impact on the performance and cyclability of the conversion-type anode. Nanoscale morphologies have the potential to achieve long cycling lifetimes and good reversibility as stress management and formation of a stable passivation layer during cycling can be achieved.

## Conclusions and outlook

Selective crystallization of electrode materials into nanostructures has presented the opportunity to design novel energy-storage materials for the next-generation, high-performance LIBs with higher energy density, higher power density, and longer cycle life. Due to the high surface area and specific configuration of nanostructured materials, these electrodes can provide high lithium-ion flux across the interface, short diffusion pathways for both Li ions and electrons, abundant active sites for Li storage, and high freedom for volume change during electrochemical charge/discharge process. In this review article, three categories of LIB electrode materials were discussed. The first one is insertion-type materials, which can store Li through an intercalation process. The improved storage ability is closely related to their surface area, crystallinity, as well as the orientation of these crystallites. In the second group, alloying-type materials such as Sn and Si were presented. Nanostructuring these bulk materials into nanowire arrays and dispersing these elements into rigid matrices have been proven to be effective approaches to overcome the poor cycling problems. The third category is conversion-type materials. Their large-scale application is also hindered by the rapid capacity decay during charging/discharging because of the significant volume change. Nanostructures with hollow interiors and nanocomposites have been developed to address this problem. For a specific material, it is hard to achieve a structure which own all advantaged features, and for different materials, the effect of the crystallization feature is not identical; therefore, much necessary work still needs to be done to give a more comprehensive understanding of the relationship between nanostructures and their performances.

To realize widespread commercial applications, controlled and large-scale fabrication of nanostructures is required, in which selective crystallization should play a vital role. The future directions of electrode materials for LIBs should focus on exploring new types of lithium-ion redox couples with different electrode reaction mechanisms and designing novel structures and morphologies in order to further increase battery energy/power densities, enhance charge/discharge rate capability, improve service life and safety, and reduce the cost at the same time. The 3D nanoarchitectured cells, in which pillared anodes and cathodes are interdigitated, have already attracted much interest. Developing flexible electrodes and all-solid batteries is a strong demand for meeting the various requirements of modern gadgets, which will also be an important feature of research in future years.

## Competing interests

The authors declare that they have no competing interests.

## Authors' contributions

FL, SS, HZ, and DX conceived the review. FL drafted the manuscript. All authors read and approved the final manuscript.
